# CHARMM-GUI *Ligand Docker* for Molecular Docking with Various Docking Programs

**DOI:** 10.1021/acs.jcim.6c00111

**Published:** 2026-03-27

**Authors:** Donghyuk Suh, Gyusik Kim, Wonpil Im

**Affiliations:** 1 Department of Biological Sciences, 1687 Lehigh University, Bethlehem, Pennsylvania 18015, United States; 2 Department of Industrial Pharmacy, 34942 Dongguk University, Seoul 04620, Republic of Korea

## Abstract

Molecular docking aims to identify a biologically relevant binding pose of a ligand in the active site of a receptor. Reliable preparation of docking systems remains a key bottleneck in structure-based drug discovery, as it requires extensive preprocessing of receptor and ligand structures and careful configuration of docking parameters. To simplify this process and promote reproducibility through online docking experiments, we have developed CHARMM-GUI *Ligand Docker*, integrating four popular docking engines (AutoDock Vina, Smina, RxDock, and DiffDock) into a unified and intuitive framework for both experts and nonexperts. Users can upload and modify receptor and ligand structures, define binding sites and flexible residues, select docking programs, and execute docking calculations. The resulting docking poses can be optionally filtered using PoseBuster to remove physically implausible or geometrically strained ligand poses. Selected poses can then be seamlessly transferred to the CHARMM-GUI *High-Throughput Simulator* to generate molecular dynamics simulation systems and inputs for further refinement or free-energy evaluation. *Ligand Docker* thus provides a robust and automated platform that bridges the gap between ligand docking and simulation, ensuring that reproducible and simulation-ready systems can be prepared rapidly and conveniently for a wide range of applications in drug discovery. *Ligand Docker* is expected to serve as a valuable web-based resource that simplifies and accelerates the multistate docking setup.

## Graphical abstract

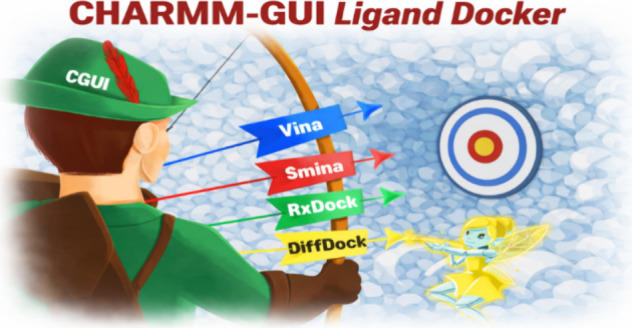

## Introduction

Molecular docking has been one of the foundational techniques in computational drug discovery for more than three decades.^1^ Its principal goal is to predict how a small molecule binds to a macromolecular target by exploring possible binding orientations, conformations, and positions of the ligand within the receptor’s binding pocket. A successful docking algorithm must efficiently sample the high-dimensional configurational space of the receptor–ligand complex and accurately score the resulting poses to select true binding poses with high binding affinities. Despite its conceptual simplicity, docking remains challenging because of the delicate balance among search efficiency, scoring accuracy, and biophysical realism.

Traditional molecular docking approaches can be viewed as a combination of a search algorithm and a scoring function.^2^ The search stage explores ligand orientations and conformations using systematic or stochastic strategies, including molecular dynamics, Monte Carlo sampling, simulated annealing, genetic algorithms, and, more recently, machine-learning-driven searches, whereas the scoring stage evaluates the resulting poses using empirical, physics-based, or knowledge-based potentials. Early scoring functions were largely derived from molecular mechanics force fields, such as CHARMM,^3–5^ AMBER,^6^ and OPLS,^7^ or from statistical analyses of known protein–ligand complexes, including DRUG-SCORE,^8^ IT-Score,^9^ and ChemScore.^10^ Empirical models, such as those implemented in AutoDock Vina^11^ and RxDock,^12^ combine van der Waals, electrostatic, hydrophobic, and hydrogen-bonding terms into weighted functions calibrated against experimental data. More recently, deep-learning-based approaches, exemplified by DiffDock,^13^ have emerged by learning complex receptor–ligand spatial distributions directly from large protein–ligand structural datasets. These developments have led to the creation of more than 60 docking programs over the past several decades, substantially improving pose prediction performance, and widely used platforms such as AutoDock-GPU^14^ and DOCK6 ^15^ are routinely employed in practical docking studies.^16^ Nevertheless, the preparation of consistent receptor and ligand inputs, binding-site definitions, and file formats remains a major source of errors and nonreproducibility across workflows.

Most docking workflows still require substantial manual intervention. Receptor preparation often involves removing crystallographic artifacts, modeling missing residues, and defining the binding-site region, while ligand preparation requires the generation of 3D geometries that can differ from force field and protonation assignments. Different docking programs accept certain file formats (pdb, pdbqt, mol2, SDF, etc.), which can introduce discrepancies during file format conversions. Differences in how these steps are performed across docking programs can lead to inconsistencies that complicate the downstream comparison or simulation refinement. Moreover, converting docking results into simulation-ready formats for molecular dynamics (MD) often requires additional scripting and parameter translation, which can be particularly time-consuming and error-prone for nonexpert users.

Since its inception in 2006, CHARMM-GUI (CGUI) has become an important resource for the molecular modeling and simulation community, providing a platform for complex molecular simulation setups through its freely accessible web-based cyberinfrastructure.^17^ Here, we introduce CGUI *Ligand Docker* (https://www.charmm-gui.org/input/ligdock), a new module designed to automate the entire receptor–ligand docking setup in a single, reproducible workflow. Built upon the modular and extensible CGUI infrastructure, *Ligand Docker* provides a user-friendly web interface that integrates receptor preprocessing, ligand generation and modification, binding-site specification, docking execution, and postdocking visualization and evaluation. The module supports four complementary docking engines: AutoDock Vina,^11^ Smina,^18^ RxDock,^12^ and DiffDock,^13^ covering a wide methodological spectrum from classical empirical scoring to modern deep-learning-based prediction. Optional PoseBuster^19^ filtering allows users to automatically detect and exclude physically implausible poses, improving downstream interpretability and reliability. To facilitate effective use, we provide a tutorial video to demonstrate key functionalities of *Ligand Docker* (https://www.charmm-gui.org/demo/ligdock).

Beyond docking itself, *Ligand Docker* connects directly with CGUI *High-Throughput Simulator* (HTS),^20,21^ enabling selected docked complexes to be prepared for all-atom MD simulations. This tight integration bridges the gap between ligand docking and simulation, allowing users to refine and validate docking results under fully atomistic and physically consistent conditions. By combining automated structure preparation, diverse docking engines, and direct simulation coupling, *Ligand Docker* provides a robust and reproducible environment for both exploratory virtual screening and detailed structure-based mechanistic studies.

## Methods

### Workflow of *Ligand Docker*

The overall *Ligand Docker* workflow is summarized in Figure 1. It begins with the *PDB Reader and Manipulator*,^22–24^ which allows users to retrieve structures directly from the RCSB database, AlphaFold DB,^25,26^ or local uploads. Users can select specific molecular components (proteins, ligands, RNA, waters) and perform structural edits such as adding covalent ligands, modeling missing residues, introducing mutations, adjusting protonation states, forming disulfide bonds, incorporating glycosylation and other post-translational modifications, etc.

**Figure 1:**
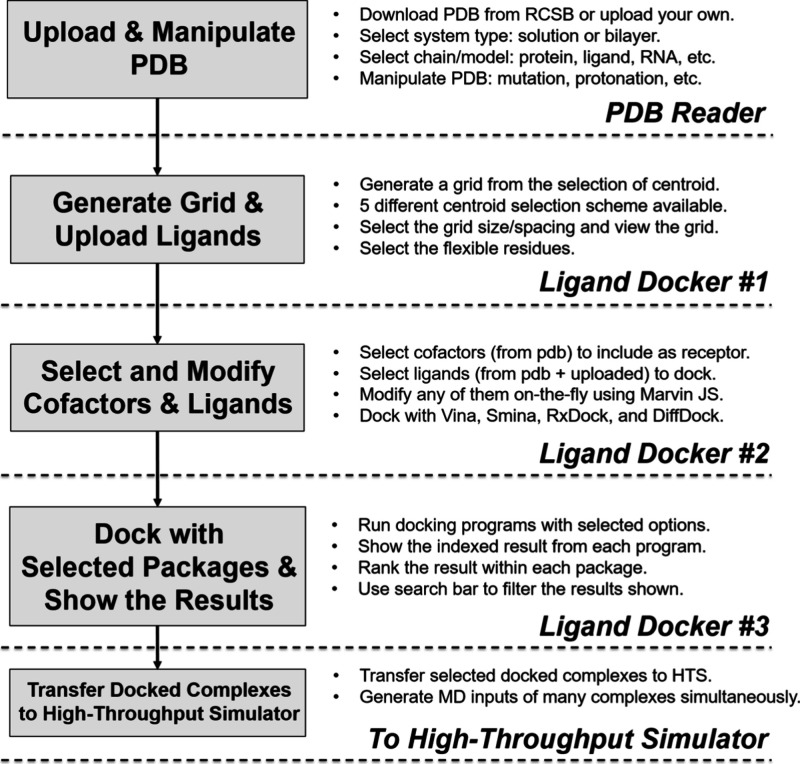
Schematic workflow of *Ligand Docker.*

Next, docking grid generation (Figure S1A) defines the binding region. Users may select a bound ligand from the PDB file and use its center of mass as the grid center. For apo structures, users can choose from four alternatives: residue-based manual selection, 3D radius-based selection, automated site prediction via VisGrid, or blind docking (full-space docking that is used for DiffDock). Grid size and resolution can then be customized, and nearby residues may be designated to be flexible for induced-fit docking (IFD). All grid and flexibility settings are displayed interactively for confirmation. Additional ligands for docking can be uploaded in the SDF format.

On the setup page (Figure S1B), the cofactor and ligand modification step provides real-time editing through integrated 3D and 2D interfaces. Selected cofactors appear in the NGL viewer,^27^ and the “Modify” option launches the Marvin JS^28^ editor for direct chemical editing. Utilizing the functions from *Ligand Designer* ^29^ and *QM/MM Interfacer*,^30^ modified structures are aligned using one of three maximum common substructure (MCS) matching schemes: element + bond order, element-only, or atom-level matching. All hetero chains in the PDB file are automatically added to the ligand list for optional self-docking, and unwanted entries can be removed. At the bottom of the page, users can choose one or more docking engines among Vina, Smina, RxDock, or DiffDock, with program-specific options including sampling number controls for all engines, maximum energy difference thresholds for Vina and Smina, and selectable scoring functions for Smina (with the commonly used “default” scoring function preselected). Users can decide to use the PoseBuster filter here to screen out poor-quality poses after docking. *Ligand Docker* visualizes results that passed all the following PoseBuster criteria: sanitization, all-atom connection, aromatic ring flatness, double bond flatness, and volume overlap with protein.^19^

After computation, result visualization (Figure S1C) presents all docked poses with ligand and cluster IDs, docking scores, and ranking. The search bar at the top allows users to filter the results by the specific program, ligand, rank, or any combinations. Individual complexes can be inspected interactively, and the entire job directory is downloadable for postprocessing, or selected docking poses can be sent to HTS for high-throughput simulation input generation.

The seamless transition from docking to MD through HTS enables users to apply MD refinement selectively, rather than uniformly, to maximize cost-effectiveness. In practice, MD refinement is most valuable when (i) no reliable reference ligand pose is available to validate docking hypotheses, (ii) docking produces multiple competing clusters without a clear consensus, (iii) the target binding site is known or suspected to be flexible, (iv) the ligand contains torsionally complex motifs or charged/polar groups whose interactions are sensitive to solvent and protonation, or (v) docking ranks are tightly packed such that physics-based relaxation can improve discrimination. Conversely, if a high-confidence reference ligand and a stable pose cluster are available and docking results are strongly converged, then users may prioritize short MD only for sanity checks and proceed directly to downstream scoring for the top-ranked subset.

## Supported Docking Programs

### AutoDock Vina and Smina

AutoDock Vina and Smina use semiempirical scoring functions that approximate the binding free energy as a weighted combination of simple interaction terms:

$$E_{\mathrm{Vina}/\mathrm{Smina}}=\omega_{1}E_{\mathrm{gauss},1}+\omega_{2}E_{\mathrm{gauss},2}+\omega_{3}E_{\mathrm{repulsion}}+\omega_{4}E_{\mathrm{hydrophobic}}+\omega_{5}E_{\mathrm{hydrogen}}$$

where $E_{\mathrm{gauss},1}$ and $E_{\mathrm{gauss},2}$ model attractive van der Waals complementarity, $E_{\mathrm{repulsion}}$ penalizes steric overlap, $E_{\mathrm{hydrophobic}}$ accounts for hydrophobic burial, and $E_{\mathrm{hydrogen}}$ rewards hydrogen-bonding geometry.^11,31^ The empirical coefficients $\omega_{i}$ are fitted to experimental binding data. The predicted binding affinity is expressed as

$$\Delta G_{\mathrm{bind}}\approx E_{\mathrm{inter}}+E_{\mathrm{intra}}+\Delta G_{\mathrm{tors}}$$

where $E_{\mathrm{inter}}$ and $E_{\mathrm{intra}}$ correspond to inter- and intramolecular interaction energies, respectively, and $\Delta G_{\mathrm{tors}}$ penalizes torsional entropy associated with ligand flexibility.

Though AutoDock Vina and Smina share this functional form in terms of scoring, they differ in implementation flexibility and parametrization philosophy. While AutoDock Vina emphasizes efficiency and simplicity through a fixed scoring function and an efficient stochastic search algorithm for robust general-purpose docking, Smina allows user-defined reparameterization of scoring terms, enabling customized weights and additional interaction functions.^32^

### RxDock

The RxDock potential combines physical and knowledge-based terms within a modular energy framework:

$$E_{\mathrm{RxDock}}=E_{\mathrm{inter}}+E_{\mathrm{intra}}+E_{\mathrm{site}}+E_{\mathrm{restraint}}$$

where $E_{\mathrm{inter}}$ is the main term of interest, representing the receptor–ligand interactions, and $E_{\mathrm{intra}}$ accounts for the relative energy of the ligand conformations.^12^ The site-specific term $E_{\mathrm{site}}$ describes the relative energy of the flexible regions of the binding pocket, while $E_{\mathrm{restraint}}$ is a collection of nonphysical restraint functions that can be used to bias the docking. Optimization proceeds via a combination of genetic algorithms, Monte Carlo sampling, and local minimization, ensuring diverse exploration of low-energy configurations.

### DiffDock

DiffDock departs from traditional scoring-based approaches by using a diffusion generative model to directly sample ligand poses from a learned probability distribution conditioned on the receptor. DiffDock frames docking as sampling from a conditional distribution over ligand poses, where docking degrees of freedom (translation, rotation, torsion) define a manifold. A diffusion process is defined in this space, and a neural network learns the reverse denoising updates.^33^

The forward diffusion process gradually adds Gaussian noise to a true ligand pose $x_{0}$ :

$$q\left(x_{t}|x_{t-1}\right)=N\left(x_{t};x_{t-1}\sqrt{1-\beta_{t}},\beta_{t}I\right)$$

where the forward diffusion transition kernel q is Markovian by construction and $\beta_{t}$ controls the variance schedule. During inference, a neural network $\epsilon_{\theta }\left(x_{t},t,R\right)$ conditioned on receptor features R learns to reverse this process:

$$x_{t-1}=\frac{1}{\sqrt{1-\beta_{t}}}\left(x_{t}-\frac{\beta_{t}}{\sqrt{1-{\mathrm{a̅}}_{t}}}\epsilon_{\theta }\left(x_{t},t,R\right)\right)+\sigma_{t}z$$

with a cumulative noise decay factor ${\mathrm{a̅}}_{t}$ , a noise scale factor $\sigma_{t}$ , and Gaussian noise z∼N(0,I) .^13^ The iterative denoising reconstructs high-confidence binding poses directly from learned distributions, eliminating the need for explicit energy minimization. DiffDock thus captures subtle geometric correlations between receptors and ligands while providing confidence estimates derived from its learned likelihood landscape.

### Datasets

To compare the pose prediction performance of the four docking engines integrated into CGUI *Ligand Docker* (AutoDock Vina, Smina, RxDock, and DiffDock) under different levels of difficulty, three complementary benchmark datasets were employed in this study. Specifically, the datasets include: (i) a cross-docking benchmark in which induced-fit effects arising from receptor–ligand mismatch are prominent (SCH),^21,34^ (ii) a standard core benchmark with well-defined structural quality and curation criteria (CASF-2016/PDBbind core set),^35^ and (iii) a redocking benchmark composed of high-quality cocrystal complexes released after 2021, designed to reduce potential overlap with commonly used training datasets (PoseBusters Benchmark set).^19^

These datasets were selected to evaluate *Ligand Docker* under complementary and practically relevant conditions: SCH assesses robustness in cross-docking with noncognate receptor conformations, CASF-2016 provides a curated and standardized self-docking benchmark, and PoseBusters comprises post-2021 PDB complexes to reduce potential training-set overlap. Each dataset has its own constraints that are considered in interpretation; SCH emphasizes robustness rather than absolute cognate accuracy, CASF-2016 represents an idealized self-docking setting, and PoseBusters improves temporal independence. To reduce benchmark-level leakage risk, we confirmed that there were no shared PDB IDs between PoseBusters and either SCH or CASF-2016. DiffDock was used with the publicly available pretrained model without retraining, and all engines were evaluated under identical *Ligand Docker*-generated inputs and a unified protocol.

### SCH Cross-Docking Set

The SCH cross-docking set derived from the Schrödinger IFD-MD study consists of 258 protein–ligand cross-docking pairs, spanning 41 protein targets and 199 ligands.^21,34^ In this dataset, ligands are docked into noncognate receptor structures, rather than their corresponding cocrystal receptors, thereby introducing substantial receptor–ligand mismatch. This dataset was used to examine the performance of docking engines under cross-docking conditions, where binding-site flexibility and induced-fit effects play a significant role in determining the predicted binding poses.

### CASF-2016 Dataset

The CASF-2016 dataset is a widely used benchmark constructed from the PDBbind refined set (v.2016) and comprises 285 high-quality protein–ligand complexes.^35^ The complexes included in this dataset are subject to strict structural and data curation criteria, ensuring reliable cocrystal geometries and binding annotations. In this study, CASF-2016 was used to assess docking performance under self-docking conditions using experimentally determined protein–ligand complex structures with well-established structural quality.

### PoseBusters Benchmark Set

The PoseBusters benchmark set consists of 308 protein–ligand cocrystal complexes selected from the Protein Data Bank, all of which were released after 2021.^19^ By restricting the dataset to recently published structures, this benchmark was designed to minimize potential overlap with datasets commonly used for training conventional and deep learning-based docking methods, such as the PDBbind General Set v2020. In this study, the PoseBusters benchmark set was employed as an additional self-docking benchmark for a complementary evaluation of pose prediction performance under conditions with reduced training data bias.

## Computational Details

### Benchmark Docking Calculations

Docking was conducted on three benchmark datasets using input files generated by *Ligand Docker*. For each case, holo receptor structures containing a bound reference ligand were used as an input. For AutoDock Vina, Smina, and RxDock, the docking grid was centered at the center of mass (COM) of the reference ligand with a grid size of 20 Å and a grid spacing of 0.375 Å, whereas DiffDock was executed in a blind docking mode without an explicitly defined grid. For each ligand, up to 10 poses were generated and ranked (Top 1–10), and the PoseBuster filter was enabled for all runs, so that structural plausibility checks were reported alongside the docking outputs. Importantly, pose prediction accuracy was evaluated exclusively on poses that passed all five PoseBuster criteria, with any pose failing a single criterion removed from consideration. As a result, the success rates reported in this study are expected to be more stringent and may differ from previously published benchmarks, in which such physical validity filtering was not applied. Pose prediction accuracy was evaluated using the Top-*K* success rate (*K* = 1–10) computed on the PoseBuster-passing poses only. A docking case was counted as successful at rank *K* if any of the Top-*K* poses achieved a ligand heavy-atom RMSD of ≤2.5 Å relative to the reference ligand pose. A confidence-score receiver operating characteristic (ROC) analysis was performed for DiffDock.

### Docking-to-HTS/MD Case Study

A representative docking-to-simulation case study using the PDB ID 1PXN (CDK2) from the CASF-2016 (PDBbind core) set was performed. Docking for the case study was performed using Smina, and the resulting top-ranked poses were used as starting structures for subsequent analyses. For the redocked poses, preservation of the key interactions reported for the crystal binding mode was assessed by visual inspection using Discovery Studio Visualizer (Dassault Systèmes). Each redocked pose was subsequently used as an independent starting structure for explicit solvent MD simulations, and three independent replicas were performed for each starting pose. MD systems were prepared using CGUI HTS connected to *Ligand Docker* with CHARMM36m^36^ for the protein and GAFF^37^ for the ligand. The complex was solvated with a 10 Å solvent box margin and neutralized/ionized to 0.15 M KCl, and simulations were performed at 310.15 K. Hydrogen mass repartitioning was applied to enable a 4 fs integration time step, and all simulations were run using GROMACS^38^ for 100 ns per replica.

## Results and Discussion

### Benchmark Docking Performance across Datasets

Figure 2 presents the Top-*K* (*K* = 1–10) success rate for each dataset, allowing a direct comparison of how success rates change as more top-ranked poses are considered. Across all datasets, success rates increase with increasing *K*, with the largest improvements typically observed from Top-1 to Top-3 or Top-5, followed by more gradual gains at higher *K*. This behavior indicates that, even when the highest-ranked pose fails, near-native poses are often present among the upper-ranked solutions. In both the SCH cross-docking and CASF-2016/PDBbind core sets, DiffDock achieves the highest Top-1 success rates, whereas AutoDock Vina and Smina exhibit steady improvements as *K* increases; RxDock shows consistently lower success rates across the entire *K* range. In contrast, in the PoseBusters benchmark set, DiffDock exhibits reduced performance relative to the other two datasets, while AutoDock Vina and Smina outperform the other engines.

**Figure 2:**
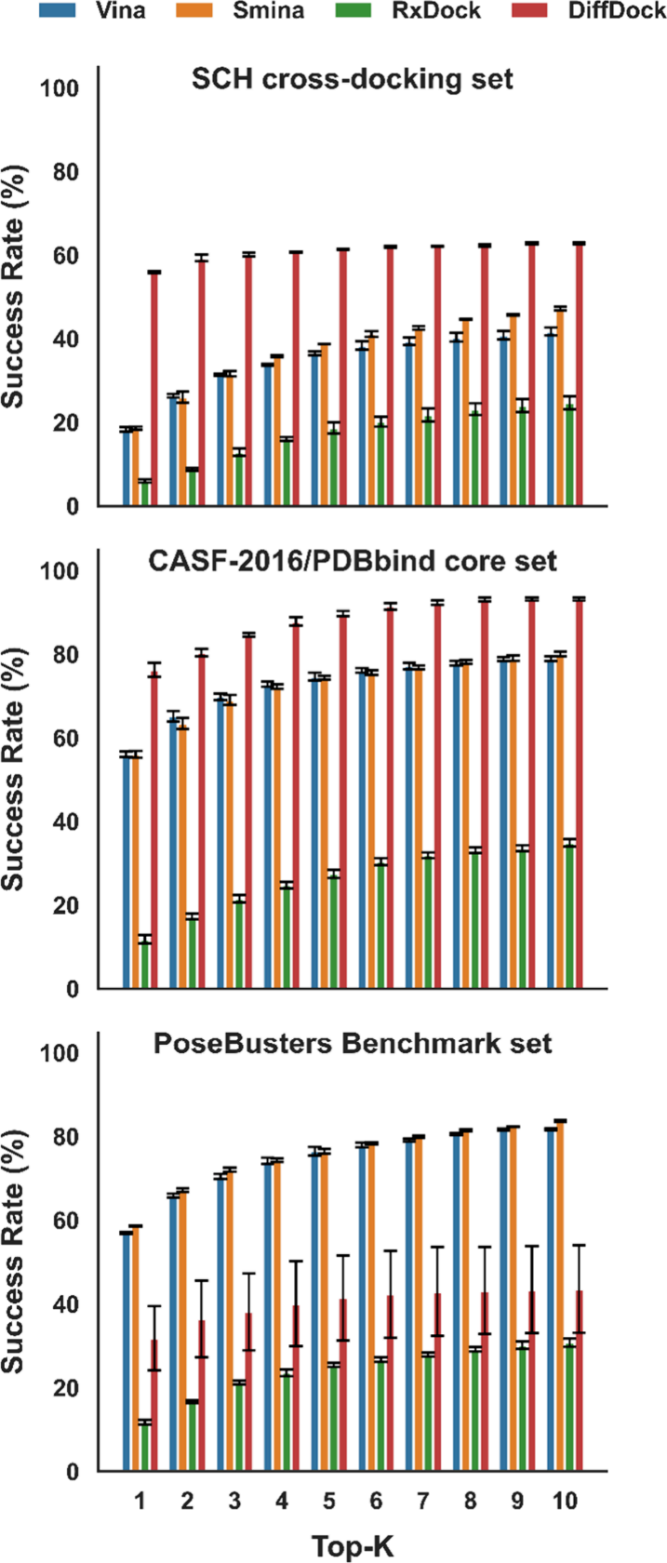
Top-*K* pose prediction success rates (*K* = 1–10) for the SCH cross-docking set, the CASF-2016/PDBbind core set, and the PoseBusters benchmark set. A prediction was considered successful at *K* when at least one of the Top-*K* poses achieved a ligand heavy-atom RMSD ≤ 2.5 Å to the reference pose. Bars show the mean success rate, and error bars indicate standard error (SE) over three independent repeats.

Taken together, these trends indicate that the relative contributions of pose sampling and pose ranking differ across docking engines and dataset types. In the SCH cross-docking set, where receptor–ligand mismatch and induced-fit effects are expected to be more pronounced, the gradual improvement of AutoDock Vina and Smina with increasing *K* suggests that near-native poses are frequently sampled but not consistently assigned to the top rank. In contrast, DiffDock’s strong Top-1 performance in the first two datasets and its comparatively smaller gains at higher *K* indicate that its predictions are more concentrated among the highest-ranked poses. The reduced performance of DiffDock on the PoseBusters benchmark set further suggests increased sensitivity to dataset characteristics, including structural novelty and the strict application of physical plausibility filtering of ligand poses. To further assess the interpretability and practical utility of DiffDock predictions, we analyzed whether the model confidence score predicts Top-1 pose success. ROC analysis shows strong discrimination across the SCH cross-docking, CASF-2016/PDBbind core, and PoseBusters benchmark sets, with replicate-averaged area under the ROC curve (AUC) values of 0.944, 0.876, and 0.856, respectively (Figure S2). Youden-index analysis yields dataset-specific optimal confidence cutoffs of −1.663, −1.300, and −1.263, respectively, providing practical confidence thresholds for enriching accurate DiffDock poses when no experimental reference pose is available.^39^

The performance trends observed for DiffDock and RxDock can be rationalized by considering the interaction between engine-specific methodological assumptions and the standardized docking and filtering protocols employed in this study. As a data-driven generative model, DiffDock performs well for systems that are closer in distribution to its training data. For underrepresented or novel complexes, it may generate poses with acceptable global placement yet local geometric or physicochemical inconsistencies, leading such poses to fail one or more PoseBuster criteria and be excluded from Top-*K* evaluation. In contrast, RxDock exhibited consistently lower success rates across all datasets, which likely reflects its reliance on a modular scoring framework and sampling strategy that are most effective when combined with user-defined restraints or more extensive treatment of binding-site flexibility. The intentionally standardized testing protocol used in this study, together with stringent physical plausibility filtering, may therefore disadvantage RxDock relative to other methods optimized for rigid-receptor docking. Overall, these results highlight how differences in training data coverage, sampling strategy, and postdocking filtering can strongly influence apparent docking performance under unified benchmarking conditions.

### Key Findings (Benchmarking)

Across datasets, Top-*K* evaluation (especially *K* = 3–5) recovers substantial additional successes beyond Top-1, indicating that near-native poses are often present among upper-ranked solutions even when the top pose fails. DiffDock excels at Top-1 on two datasets but shows greater sensitivity to dataset novelty/filters, whereas Vina/Smina benefit more from increasing *K*, consistent with good sampling but imperfect ranking under standardized conditions. For DiffDock, confidence-score ROC analysis further showed strong discrimination of the Top-1 pose success.

### Docking-to-HTS/MD Case Study: Pose Stability and Reranking

To directly examine whether docking rank reliably identifies a stable binding pose and to explore whether MD-based postprocessing can provide additional discrimination, we performed a representative docking-to-simulation case study using PDB ID 1PXN from the CASF-2016/PDBbind core set. The 1PXN crystal structure is a human CDK2–inhibitor complex with the bound ligand 4-[4-(4-methyl-2-methylamino-thiazol-5-yl)-pyrimidin-2-ylamino]-phenol (Figure S3A).^40^ To evaluate docking poses beyond the docking score, we examined whether each pose satisfied key interactions observed in the crystal binding mode: (i) hinge hydrogen bonding to Glu81/Leu83, (ii) a directional polar contact involving Lys89, and (iii) hydrophobic packing against Phe80/Leu134. These interactions, as illustrated in Figure S3A, are used to compare representative docking poses.

Figures S3B and S3C show the Top-1 and Top-2 docking poses, respectively, positioned in the same binding site for direct comparison with the crystal reference; all Top-10 docking scores and ligand heavy-atom RMSDs are shown in Figure 3. As illustrated in these panels, the Top-1 docking pose deviates substantially from the crystal binding mode, whereas the Top-2 pose more closely maintains the key interaction pattern. This discrepancy is also reflected in the quantitative docking metrics. As shown in Figure 3A, the Top-1 pose achieves the most favorable docking score, yet Figure 3B reveals that it exhibits a large ligand heavy-atom RMSD relative to that of the crystal structure (6.60 Å). In contrast, the Top-2 pose shows the lowest RMSD (1.06 Å), indicating superior structural agreement with the crystal binding mode. Together, these results demonstrate that docking rank alone may not reliably capture structural plausibility, and that the top-scoring pose is not necessarily the most appropriate starting structure for downstream simulations.

**Figure 3:**
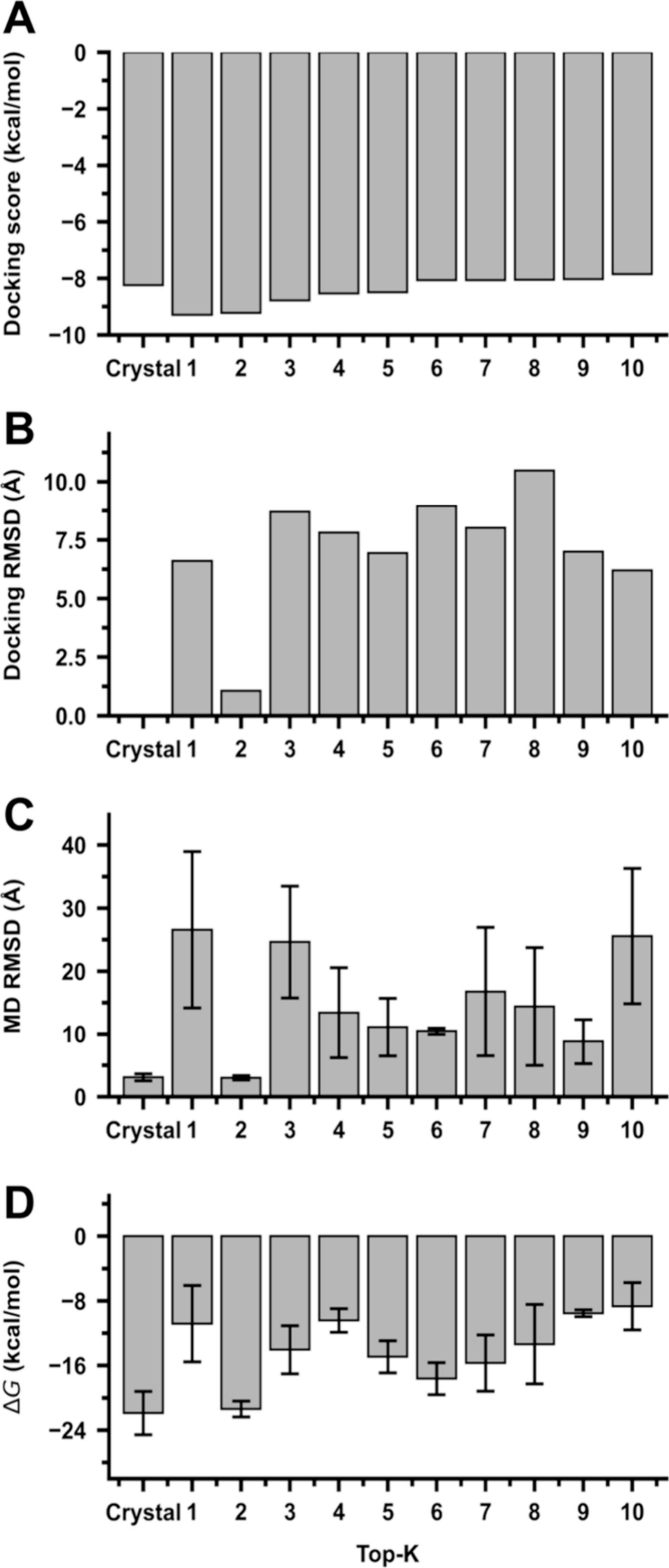
Quantitative evaluation of docking poses for the CDK2–inhibitor complex (PDB ID 1PXN). (A) Docking scores of the crystal reference (rescored crystal binding pose) and top-ranked docking poses. (B) Ligand heavy-atom RMSD relative to the crystal reference structure. (C) Average ligand heavy-atom RMSD during the last 10 ns of MD simulations, computed after protein alignment. (D) MM/GBSA binding free-energy estimates (Δ*G*) averaged over MD snapshots. While the Top-1 pose achieves the most favorable docking score, the Top-2 pose exhibits superior structural stability and more favorable binding energetics during MD, highlighting the added value of MD-based postprocessing for pose discrimination.

MD-based analyses provide additional discrimination beyond these static docking measures. As shown in Figure 3C, the crystal reference and the Top-2 pose maintain a low average ligand heavy-atom RMSD during the last 10 ns of MD simulations, consistent with stable binding geometries. In contrast, the Top-1 pose and several lower-ranked poses exhibit markedly higher RMSD values, suggesting reduced pose stability under dynamic conditions. This stability trend is further supported by MM/GBSA binding free-energy estimates (Figure 3D), which indicate more favorable binding energetics for the crystal reference and the Top-2 pose compared to those of the Top-1 pose. Taken together, these results demonstrate that docking rank alone is insufficient to identify the most structurally stable and energetically favorable binding pose. Instead, MD-based postprocessing that incorporates trajectory-based stability and binding free-energy assessments can reveal meaningful differences among top-ranked docking poses, thereby providing a more robust basis for postdocking pose prioritization.

### Key Findings (Docking to MD)

Docking score/rank alone can mis-prioritize poses. In the 1PXN case, the Top-1 pose scores best yet is structurally incorrect and unstable, whereas the Top-2 pose is near-native and remains stable in explicit solvent. MD-based refinement and trajectory-based scoring (RMSD stability, MM/GBSA) provide orthogonal information that can rerank the top docking candidates and improve pose prioritization when docking ranks are ambiguous.

Overall, the benchmark analysis indicates that Top-*K* evaluation captures meaningful performance gains beyond Top-1 across diverse dataset conditions. In this context, the 1PXN case study further demonstrates that MD-based stability and energetic assessments can reveal differences among top-ranked docking poses that are not apparent from the docking rank alone. Although the HTS/MD analysis presented here is limited to a single representative complex and a single docking engine (Smina), it serves as a practical example of how MD refinement and trajectory-based scoring can be integrated into practical workflows to further refine and prioritize docking outputs (see also refs 20 and 21).

## Conclusions

In this work, we introduce CGUI *Ligand Docker*, a web-based module that provides a unified and reproducible framework for receptor–ligand docking within the CGUI ecosystem. By integrating widely used multiple docking engines, including AutoDock Vina, Smina, RxDock, and DiffDock, *Ligand Docker* enables users to carry out docking calculations using diverse methodological paradigms while maintaining consistent receptor and ligand preparation, binding-site definition, and output formatting. In the current release, these engines operate independently by design to support modular benchmarking and transparent comparison. As future work, we plan to implement consensus scoring strategies that integrate results across engines to further improve robustness and ranking performance, as well as optional interaction fingerprint profiling of hydrogen bonds and key contacts to complement RMSD- and rank-based pose evaluation.

Benchmark calculations on cross-docking and self-docking datasets demonstrate that *Ligand Docker* reliably supports these docking engines under a standardized workflow and that pose recovery improves substantially when multiple top-ranked poses are considered rather than relying solely on the highest-scoring solution. The inclusion of PoseBuster filtering further improves the interpretability of docking results by identifying and removing physically implausible poses prior to downstream analysis. A representative docking-to-molecular dynamics case study illustrates that docking rank alone does not necessarily correlate with pose stability or binding energetics under explicit solvent simulations. The direct integration of *Ligand Docker* with the HTS enables rapid preparation of simulation-ready systems, providing a practical route for MD-based refinement, stability assessment, and postdocking reranking of candidate poses.

Overall, *Ligand Docker* lowers technical barriers associated with docking setup and postprocessing, promotes reproducibility across docking workflows, and facilitates seamless extension of docking results to atomistic simulations. The module is expected to serve as a useful and accessible resource for structure-based modeling, virtual screening, and mechanistic studies of computational drug discovery.

## Supplementary Material



## Data Availability

The data underlying this study including the input files for model system generation are freely available in *Ligand Docker* Archive in CHARMM-GUI (http://www.charmm-gui.org/docs/archive/ligdock) and can be reproduced in CHARMM-GUI *Ligand Docker* (https://charmm-gui.org/input/ligdock).

## Notes

Additional implementation details with representative screenshots and docking analyses, including confidence-based ROC curves for DiffDock and structural comparisons of predicted binding poses (PDF)
