# Supplementary material for: CHARMM-GUI Ligand Docker for Molecular Docking with Various Docking Programs
Source: J Chem Inf Model. 2026 Mar 27;66(7):3416–23. doi: 10.1021/acs.jcim.6c00111 (PMC13080963; doi:10.1021/acs.jcim.6c00111)
Supplement: Supplementary file 1 [file ci6c00111_si_001.pdf]

Supporting Information

CHARMM-GUI *Ligand Docker* for Molecular Docking with Various Docking Programs

Donghyuk Suh,[1] Gyusik Kim,[2] and Wonpil Im[1]*

[1]Department of Biological Sciences, Lehigh University, Bethlehem, Pennsylvania 18015, USA

[2]Department of Industrial Pharmacy, Dongguk University, Seoul 04620, Republic of Korea

Corresponding Author Email: Wonpil Im (wonpil@lehigh.edu)

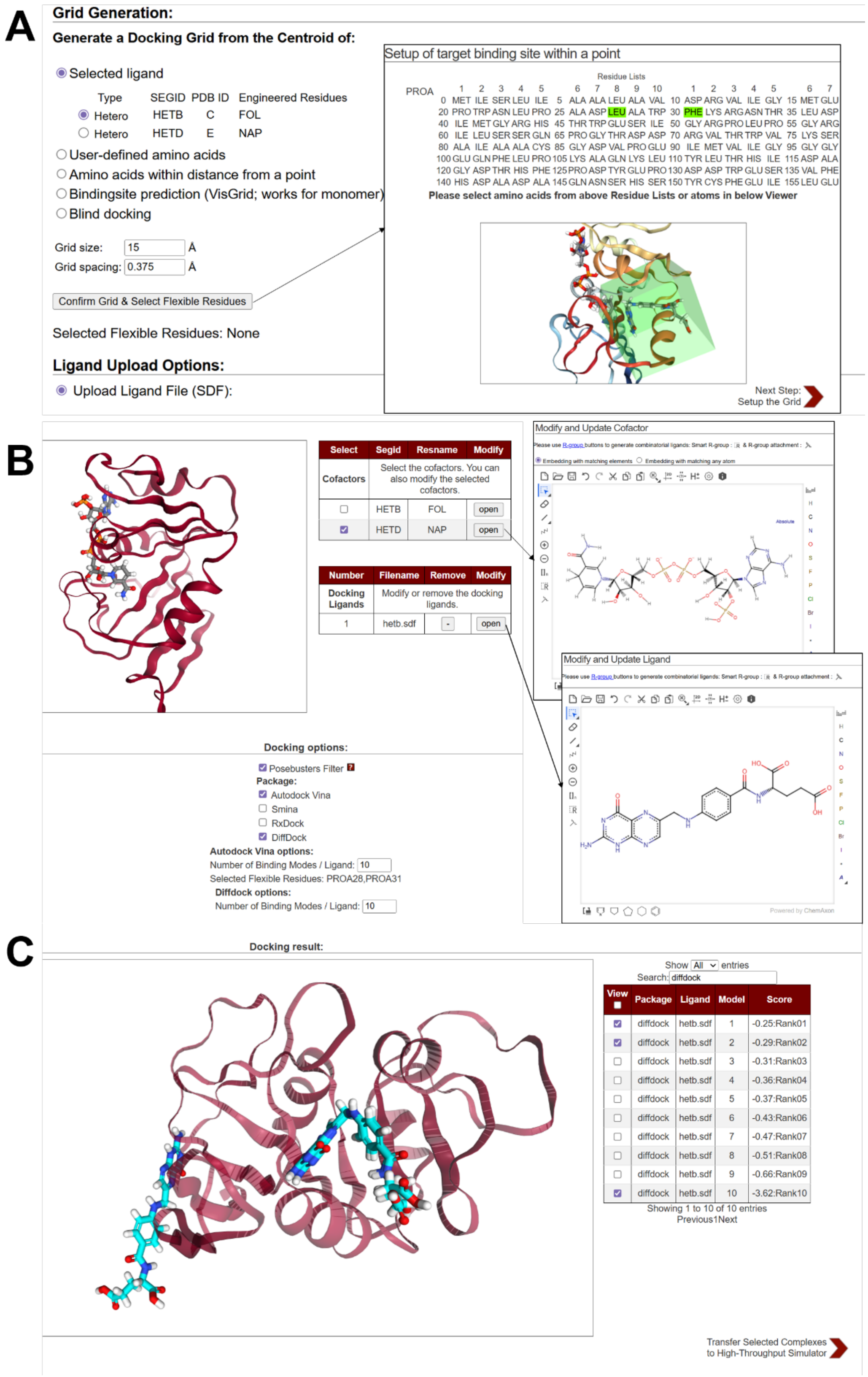

Figure S1. *Ligand Docker* implementation details. (A) Generating a docking grid with flexible residue selection and uploading additional docking ligands. (B) Modifying cofactor/ligands and docking with selected programs. (C) Showing docking results and transferring selected docked complexes to CGUI *HTS* to generate MD systems and inputs.

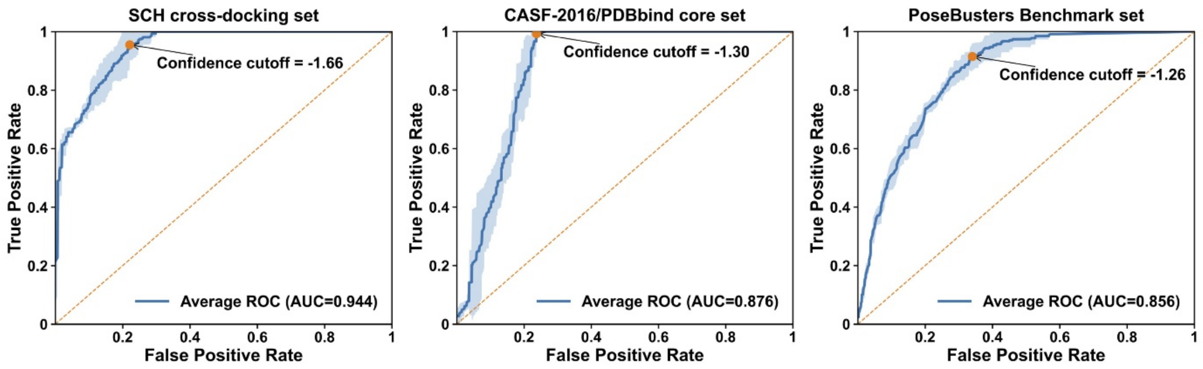

Figure S2. Confidence-based ROC curves for DiffDock across benchmark datasets (left to right: the SCH cross-docking set, the CASF-2016/PDBbind core set, and the PoseBusters benchmark set). A Top-1 prediction was considered successful when the ligand heavy-atom RMSD to the experimental pose was ≤ 2.5 Å. Solid lines indicate replicate-averaged ROC curves and shaded regions show variability across replicates. Replicate-averaged AUC values are shown in each panel. Orange markers denote the Youden-index optimal confidence cutoffs (−1.663, −1.300, and −1.263, respectively).

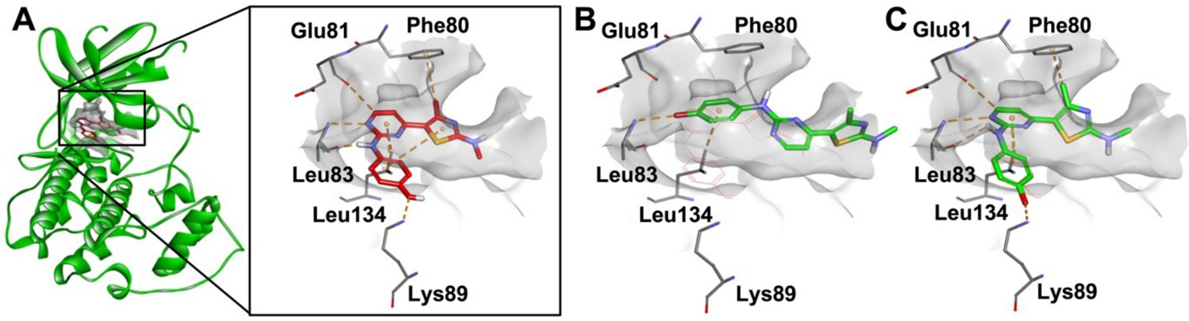

Figure S3. Structural comparison of the crystal reference binding mode and representative docking poses for the CDK2–inhibitor complex (PDB ID 1PXN). (A) Overall structure of CDK2 with the crystal reference ligand shown in the binding site, highlighting key interactions used for pose evaluation, including hinge hydrogen bonding to Glu81/Leu83, a directional polar contact involving Lys89, and hydrophobic packing against Phe80/Leu134 (dashed lines indicate hydrogen-bond/polar contacts). (B) Top-1 docking pose and (C) Top-2 docking pose shown in the same binding site for direct comparison with the crystal reference.
